# Determinants of long-acting contraceptive utilization among married women of reproductive age in Aneded district, Ethiopia: a case–control study

**DOI:** 10.1186/s13104-019-4445-3

**Published:** 2019-07-18

**Authors:** Abebe Habtamu, Mulugeta Tesfa, Mengistie Kassahun, Simachew Animen

**Affiliations:** 1 Department of Public Health, College of Health Sciences, Debre Markos University, Debre Markos, Ethiopia; 2 Department of Public Health, College of Medicine and Health Sciences, Bahir Dar University, Bahir Dar, Ethiopia; 3 Department of Midwifery, College of Medicine and Health Sciences, Bahir Dar University, Bahir Dar, Ethiopia

**Keywords:** Long-acting, Contraception, Education, Married women, Reproductive age, Aneded district health office, Ethiopia

## Abstract

**Objectives:**

To identify the determinants of long-acting contraceptive utilization among married women of reproductive age in Aneded district, northwestern Ethiopia. Unmatched case control study was conducted from May 1, 2018 to June 30, 2018. One hundred forty-five households with married reproductive age women who have used long-acting family planning for more than a year (cases) and 290 households with married reproductive age women who have never used long-acting family planning (controls) were selected by systematic random sampling in each kebele (the smallest administrative units of Ethiopia).

**Result:**

In this study, 145 cases and 290 controls participated. Independent positive predictors of utilization of long-acting family planning among married women reproductive age were: primary education level [AOR = 6.99, 95% CI 3.7–13.7], first discussion with providers [AOR = 2.64, 95% CI 1.6–4.5], told what to do if they experience the side effect [AOR = 3.2, 95% CI 1.7–5.9], know the source of long-acting family planning methods [AOR = 3.4, 95% CI 2.01–5.82] and discussion with health professionals [AOR = 2.07, 95% CI 1.2–3.5]. Encouraging women education at least at primary level and advocating the minimal side effect of long-acting contraceptive are recommended to improve long-acting family planning utilization.

## Introduction

Family planning is the capability of individuals or couples to decide the desired number of their children and the spacing and timing of births through the use of contraception. It is also a means of encouraging the health of women and families and part of a strategy to reduce maternal, infant and child mortality. Methods of family planning are broadly categorized into natural family planning and modern family planning methods. Modern methods are further categorized as short-acting and long-acting contraceptive methods. Intrauterine contraceptive devices (IUCD) and implants are long-acting temporary methods [1].

Effective contraceptive can prevent as many as one in every three maternal deaths by allowing women to delay motherhood, space births, avoid unintended pregnancies and abortions and limit childbearing when they have reached their desired family size [2].

Reproductive age women have varying contraceptive needs. Long-term reversible contraceptive is an excellent choice for majority of women. These methods can enhance family planning (FP) programs in meaningful ways if the challenges to their availability, access, and acceptability can be overcome [3].

When we compare long-acting reversible contraceptive methods (LARCM) with short-acting contraceptive methods, the long-acting ones are convenient for users and effectively prevent pregnancy, cost-effective for programs over time, can result in substantial cost savings for governments and contribute directly to reaching national and international health goals [4].

In 2012, an estimated 645 million women in the developing world were using modern methods. The percentage of married women using modern contraceptives increased from 56% in 2008 to 57% in 2012. Serving all women in developing countries that currently have an unmet need for modern methods would prevent an additional 54 million unintended pregnancies, including 21 million unplanned births, 26 million abortions and seven million miscarriages; this would also prevent 79,000 maternal deaths and 1.1 million infant deaths [5].

In Africa, utilization of long-acting family planning methods, especially IUCD and implants are very low. In this region, utilization of IUCD and implants were 4.6% and 1.0% respectively [6].

Based on the Ethiopian Demographic Health Survey (EDHS) 2016, permanent family planning utilization was less than 1%, intrauterine device 2% and women using implant 8% from 35% of the national modern contraceptive prevalence whereas in Amhara Regional State unmet need for family planning accounts about 17%, nearly equivalent to the national level [7].

Some of the obstacles in the utilization of long-acting contraceptive methods are: services or supplies are not adequately available, choices are limited, fear of social disapproval, partners’ objections, worries of side effects, health concerns, lack of knowledge about contraceptive choices and their use [8].

In countries with high fertility rate and unmet need of contraceptives, shifting towards long-acting family planning methods (LAFPMs) is an important strategy to ensure continuity of services. However, currently; modern contraceptive use is dominated by short-term methods which were reported to be as 25% with injectable being (23%) and pills (2%) among married women. Similarly, the overall prevalence of long-acting family planning in Ethiopia was 10%. However, utilization of family planning methods in countries with high birth rates and limited resources have a potential for improving maternal and child health, but the proportion of users of long-acting methods is very low in Ethiopia [7, 8].

At country level, in Ethiopia the Federal Ministry of Health has made considerable effort to improve utilization of maternal health by providing long-acting family planning methods for prevention of unwanted pregnancy and premature maternal death. Methods are available for each peripheral institute, but still we are experiencing maternal health related problems including unwanted pregnancy, infant and maternal death. Long-acting contraceptive is a single key solution for all problems of the leading cause of maternal death. Even if different contributing factors are identified, utilization of long-acting contraceptive is continuously low [9].

Previous studies focused on prevalence of long-acting family planning and methodologically the studies were cross-sectional. The objective of this study was to identify determinants of utilization of long-acting contraceptive methods among married women of reproductive age in Aneded district, north-west Ethiopia.

## Main text

### Methods

#### Study setting and period

A community based unmatched case control study was conducted from May 1 to June 30, 2018 in Aneded district, which is located north-western Ethiopia, in Amhara Region, 278 km from Addis Ababa (the capital city of Ethiopia) and 260 km from Bahir Dar, the regional capital.

The district had a total population of 108,326 of which 22,549 were women expected to be in total reproductive age group. The district is administratively divided into 19 kebeles (the smallest administrative units). There were four health centers and 19 health posts in the district with a total of 10 midwifery, 42 health extension workers, 97 health workers and 54 support staff [10].

#### Sample size and sampling techniques

The required sample size was calculated by using Epi Info version 7 with double population proportion formula. It was calculated by assumption of health extension visit as factor [11], 95% confidence interval, 80% power, 10% non-response rate, and case to control ratio 1:2 to increase the power of the study. The total sample size was 414 (138 cases and 276 controls). Then adding 5% non-response rate to each group it become 435 (145 cases and 290 controls).

*Cases* were those women who used long-acting contraception for more than a year. *Controls* were those married women of reproductive age who never used long-acting family planning methods.

The total sample size was proportionally allocated for the ten kebeles based on the number of married women of reproductive age who reside in the study area.

Households with married reproductive age women who were using long-acting family planning and households with married reproductive age women who did not use long-acting family planning were selected by systematic random sampling in each kebele for the study.

#### Measurement

Training was given for data collectors and supervisors on methods of extracting the information through interviewing, recording information generated through structured questionnaire and ways of approaching respondents. Data were collected through face to face interview method by using a structured and pre-tested questionnaire. Collected data were checked daily by supervisors for completeness, legibility and consistency.

#### Statistical analysis

Collected data were checked for completeness, coded and entered into Epi data version 3.1 and then exported to SPSS version 20 for data analysis. First bivariate analysis was done by using binary logistic regression model and all variables which were found to be significant at p-value less than 0.2 were entered into multiple logistic regression. Independent variables that were significant at p-value 0.05 levels in the multiple logistic regression model analysis were considered as determinant factors of utilization of long-acting contraceptive. The strength of association between dependent variables and independent variables were expressed in crude and adjusted odds ratio with 95% confidence interval. Finally results were presented in the form of text, tables and figures.

### Results

#### Socio-demographic characteristics

Participants enrolled into the study were 435 (145 cases and 290 controls) with a 100% response rate for both cases and controls. The median age of cases and controls were 34 and 29.6 respectively (Table 1).

**Table 1:** Socio-demographic characteristics of married women related to long-acting family planning utilization in Aneded district, Ethiopia, 2018 (n = 435)

Factors	Category	Case	Control	Total
		N (%)	N (%)	N (%)
Age of respondents	15–24	25 (17.2)	58 (20)	83 (19.1)
	25–29	28 (19.3)	128 (44.1)	156 (35.9)
	30–34	23 (15.9)	46 (15.9)	69 (15.9)
	35–39	51 (35.2)	44 (15.2)	95 (21.8)
	40–49	18 (12.4)	14 (4.8)	32 (7.4)
Religion of respondents	Orthodox	130 (89.7)	259 (89.3)	389 (89.4)
	Islam	15 (10.3)	31 (10.7)	46 (10.6)
Educational level of respondents	Cannot read and write	62 (42.8)	195 (67.2)	257 (59.1)
	Can read and write but have no formal education	4 (2.8)	9 (3.1)	13 (3)
	Primary (1–8)	79 (54.5)	86 (29.7)	165 (37.9)
Husband educational level	Cannot read and write	104 (71.7)	205 (70.7)	309 (71)
	Can read and write but have no formal education	27 (18.6)	63 (21.7)	90 (20.7)
	Primary (1–8)	14 (9.7)	22 (7.6)	36 (8.3)
Occupation of respondents	Housewife	136 (93.8)	271 (93.4)	407 (93.6)
	Merchant	9 (6.2)	19 (6.6)	28 (6.4)
Husband occupation	Farmer	134 (92.4)	265 (91.4)	399 (91.7)
	Merchant	9 (6.2)	15 (5.2)	24 (5.5)
	Government employee	2 (1.4)	10 (3.4)	12 (2.8)
Family monthly income in Ethiopian birr	< 929	37 (25.5)	48 (16.6)	85 (19.5)
	930–1300	33 (22.8)	81 (27.9)	114 (26.2)
	1301–1450	18 (12.4)	46 (15.9)	64 (14.7)
	1451–1700	30 (20.7)	60 (20.7)	90 (20.7)
	> 1700	27 (18.6)	55 (19)	82 (18.9)

#### Factors associated with utilization of long-acting family planning

Multivariate logistic regression showed that association with utilization of long-acting family planning among married women of reproductive age at p value less than 0.05 were conducted with respect to: educational status of the respondents, discussion with health professionals, knowing the source of long-acting FP, first provider discussion and told what to do if side effect is experienced due to the method.

This study revealed that those reproductive age married women who could not able to read and write were 7 times less likely to utilize long-acting family planning method as compared to those reproductive age of married women who completed their primary level of educational [AOR = 6.99, 95% CI 3.7–13.3].

Women who had discussion with the health workers (professionals) were 2 times [AOR = 2.07, 95% CI 1.2–3.5] more likely to use LACM compared to women who had no discussion.

Those reproductive age married women who know the source of long-acting family planning methods were 3 times [AOR = 3.4, 95% CI 2.01–5.82] more likely to utilize long-acting method as compared to those who did not know its source where it is found. In addition, married women of reproductive age who had discussion with providers first about the methods were two times [AOR = 2.64, 95% CI 1.6–4.5] more likely to use LACM compared to those who had no first discussion with providers about which method to use.

Reproductive age married women who were told the information about what to do if they experienced any side effect were 3 times [AOR = 3.22, 95% CI 1.7–5.91] more likely to utilize long-acting methods than those non-exposed counterparts as summarized in Table 2.

**Table 2:** Bivariate and multivariate analysis results of determinants of long-acting family planning among married women of reproductive age in Aneded district, 2018

Factors	Category	Case	Control	COR 95% CI	AOR	p-value
		N (%)	N (%)			
Age of respondents	15–24	25 (17.2)	58 (20)	1	1	
	25–29	28 (19.3)	128 (44.1)	0.51 (0.30–0.94)	0.43 (0.86–1.21)	0.078
	30–34	23 (15.9)	46 (15.9)	1.20 (0.60–2.30)	1.03 (0.52–1.54)	0.151
	35–39	51 (35.2)	44 (15.2)	2.70 (1.50–4.90)	2.3 (0.92–4.17)	0.092
	40–49	18 (12.4)	14 (4.8)	2.90 (1.30–6.90)	2.5 (0.87–5.43)	0.164
Educational level of respondents	Cannot read and write	62 (42.8)	195 (67.2)	1		
	Can read and write but have no formal education	4 (2.8)	9 (3.1)	1.4 (0.5–4.7)	2.9 (0.6–14.5)	0.181
	Primary (1–8)	79 (54.5)	86 (29.7)	2.9 (1.9–4.4)*	6.99 (3.7–13.3)*	0.000
Desired duration for next birth	Within 2 years	10 (6.9)	18 (6.2)	1.83 (0.81–4.1)	1.56 (0.62–3.21)	0.065
	After 2 years	61 (42.1)	29 (10)	6.91 (4.1–11.5)	6.02 (0.92–7.54)	0.120
	Yet not decided	74 (51)	243 (83.8)	1		
Responsible for deciding to have children	Wife	85 (58.6)	67 (23.1)	4.6 (2.9–7.1)	4.32 (0.98–9.24)	0.076
	Husband	3 (2.1)	18 (6.2)	0.6 (0.17–2.1)	0.54 (0.36–1.92)	0.181
	Jointly	57 (39.3)	205 (70.7)	1		
Discussion with husband	No	43 (29.7)	146 (50.3)	1		
	Yes	102 (70.3)	144 (49.7)	2.41 (1.57–3.7)*	1.70 (0.90–3.10)	0.084
Husband attitude	Support	54 (37.2)	14 (4.8)	11.70 (6.20–22.04)	10.82 (0.69–14.09)	0.092
	Oppose	91 (62.8)	276 (95.2)	1		
Discussion with health professional	No	69 (47.6)	190 (65.5)	1		
	Yes	76 (52.4)	100 (34.5)	2.10 (1.40–3.12)*	2.07 (1.2–3.53)*	0.007
Know source LAFP	No	40 (27.6)	211 (72.8)	1		
	Yes	105 (72.4)	79 (27.2)	7.01 (4.5–10.9)*	3.4 (2.01–5.82)*	0.000*
First discussion with provider	No	32 (22.1)	176 (60.7)	1		
	Yes	113 (77.9)	114 (39.3)	5.45 (3.5–8.6)*	2.64 (1.62–4.51)*	0.000
Told what to do if side effect is experienced	No	56 (38.6)	203 (70)	1		
	Yes	89 (61.4)	87 (30)	3.70 (2.40–5.60)*	3.22 (1.70–5.91)*	0.000

*Statistical significance (*p*-value < 0.05)

### Discussion

Educational status was one of the factors that determined long-acting contraceptive utilization in this study. Married women of reproductive age who could not read and write were less likely to use long-acting contraceptive as compared to those married women of reproductive age who completed primary education level. These findings are in line with a study conducted in Ethiopia, 2016 EDHS [12]. It is also supported by the results found from Wolaita town; southern Ethiopia [13]. The reason why education is associated with long-acting contraceptive utilization is the better educated women might have better understanding about the importance of long-acting contraceptive for themselves and for their family. Educated women might have more information about different methods or options.

The other independent factor found to be determinant for long-acting contraceptive utilization was discussion with health professionals. Reproductive age of married women who discussed with health professionals were more likely to use long-acting contraceptive method than their counterparts. This result is in line with a study conducted in Nekemte town, Oromia region; west Ethiopia [14]. This might be due to health professionals providing appropriate information about the available long-acting contraceptive methods.

First discussion with providers was also another determinant factor for long-acting contraceptive utilization in this study. Those who have not had first discussion with providers which method to use were less likely to utilize than those who had first discussion with providers. These findings are in line with the study conducted in Misha district, southern Ethiopia in 2014 [15]. This might be due to the fact that first discussion with providers about which method to use gives the respondents clear understanding and enhances their informed choice to utilize long-acting contraceptive services.

In this study, telling what to do if they experience any side effect was strongly associated with long-acting contraceptive utilization as compared to those married women of reproductive age those who were not told what to do when they experienced the side effects. This result is also supported by other recent studies [6, 13]. The possible explanation might be those women who told about the expected side effects of long-acting contraceptives could not be worried since they were counseled about side effects are temporary and would disappear after a few months.

### Conclusions

This study identifies determinants of long-acting contraceptive utilization among reproductive age of married women in Aneded district, Ethiopia. It was found that educational status, discussion with health a professional (women who had discussion with health a professional), know the source of long-acting contraceptives (women who know where long-acting contraceptive method is available), first discussion with providers (women who had first discussion with providers about which method to use) and told what to do if experienced any side effect (women who those told what to do if they were experienced any side effect) were determinants of long-acting family planning utilization in this study.

Therefore, contraceptive programmers should provide information for the community about the source of long-acting contraceptive methods to increase utilization of long-acting family planning. The Woreda Education Office should encourage women to pursue their education to at least primary school level. All health professionals should discuss about long-acting family planning with all reproductive age women who come for any reason to the health institution. The service providers should clarify the side effects related with long-acting contraceptive methods.

## Limitation of the study

There were no verifying mechanisms of educational statuses of health professionals who discuss with married reproductive age women.

## Data Availability

The data sets generated during the current study are available from corresponding author on reasonable request.

## Abbreviations

CHIS: Community Health Information System
CPR: contraceptive prevalence rate
EDHS: Ethiopian Demographic Health Survey
FP: family planning
IUCD: intra uterine contraceptive device
LAFPMs: long acting family planning method
LARCMs: long acting reversible contraceptive methods
NGO: non-governmental organization
WHO: World Health Organization
